# Update on the pharmacotherapy of cerebellar and central vestibular disorders

**DOI:** 10.1007/s00415-015-7987-x

**Published:** 2016-04-15

**Authors:** Roger Kalla, Julian Teufel, Katharina Feil, Caroline Muth, Michael Strupp

**Affiliations:** Division of Cognitive and Restorative Neurology, Department of Neurology, University Hospital Bern, Freiburgstrasse 18, 3010 Bern, Switzerland; Department of Neurology and German Center for Vertigo and Balance Disorders, University Hospital Munich, Campus Grosshadern, Munich, Germany

**Keywords:** Cerebellar ataxia, Central vestibular disorders, Vestibular migraine, Aminopyridines, Episodic ataxia type 2, Downbeat nystagmus

## Abstract

An overview of the current pharmacotherapy of central vestibular syndromes and the most common forms of central nystagmus as well as cerebellar disorders is given. 4-aminopyridine (4-AP) is recommended for the treatment of downbeat nystagmus, a frequent form of acquired persisting fixation nystagmus, and upbeat nystagmus. Animal studies showed that this non-selective blocker of voltage-gated potassium channels increases Purkinje cell excitability and normalizes the irregular firing rate, so that the inhibitory influence of the cerebellar cortex on vestibular and deep cerebellar nuclei is restored. The efficacy of 4-AP in episodic ataxia type 2, which is most often caused by mutations of the PQ-calcium channel, was demonstrated in a randomized controlled trial. It was also shown in an animal model (the tottering mouse) of episodic ataxia type 2. In a case series, chlorzoxazone, a non-selective activator of small-conductance calcium-activated potassium channels, was shown to reduce the DBN. The efficacy of acetyl-DL-leucine as a potential new symptomatic treatment for cerebellar diseases has been demonstrated in three case series. The ongoing randomized controlled trials on episodic ataxia type 2 (sustained-release form of 4-aminopyridine vs. acetazolamide vs. placebo; EAT2TREAT), vestibular migraine with metoprolol (PROVEMIG-trial), cerebellar gait disorders (sustained-release form of 4-aminopyridine vs. placebo; FACEG) and cerebellar ataxia (acetyl-DL-leucine vs. placebo; ALCAT) will provide new insights into the pharmacotherapy of cerebellar and central vestibular disorders.

## Introduction

In this overview, the current pharmacological treatment of central vestibular vertigo, cerebellar disorders and the most common forms of central nystagmus are presented. These disorders are usually associated with vertigo and dizziness, which are among the most frequent symptoms with which patients present to primary care physicians with a 30 % lifetime prevalence [1]. The general prerequisites for successful treatment of dizziness and nystagmus are a correct diagnosis, the correct drug, an appropriate dosage, and a sufficient duration of the treatment [5]. Vertigo and dizziness due to central vestibular disorders are often associated with nystagmus, which causes blurred vision and impaired visual acuity due to oscillopsia. Depending on the underlying etiology, additional cerebellar and brain stem signs may occur [2]. In recent years, progress has been made in the symptomatic treatment of cerebellar disorders such as episodic ataxia type 2 (EA2) as well as downbeat nystagmus (DBN), in particular due to the use of aminopyridines [3, 4].

Non-randomized and non-placebo-controlled clinical trials on the treatment of different forms of dizziness and nystagmus are not state-of-the-art and have considerable deficits. Thus, there is a strong need for multicenter, placebo-controlled trials for vestibular and ocular motor disorders. The ongoing trials in the field are also described (Table 1) and perspectives for future therapeutic interventions are discussed.

**Table 1:** Current ongoing randomized clinical trials for central vestibular disorders at the German Center for Vertigo, Munich. A, B, and C are funded by the Federal Ministry of Research

(A) Vestibular migraine	Metoprolol (95 mg/d) versus placebo: PROVEMIG-trial
(B) Episodic ataxia type 2	Fampridine (20 mg/d) versus acetazolamide (750 mg/d) versus placebo: EAT-2-TREAT-trial
(C) Cerebellar ataxia	Acetyl-DL-leucine (5 g/d) versus placebo: “ALCAT-trial”
(D) Cerebellar gait ataxia	Fampridine (20 mg/d) versus placebo: “FACEG-trial”

### Downbeat, upbeat nystagmus and other forms of nystagmus

**Fig. 1:**
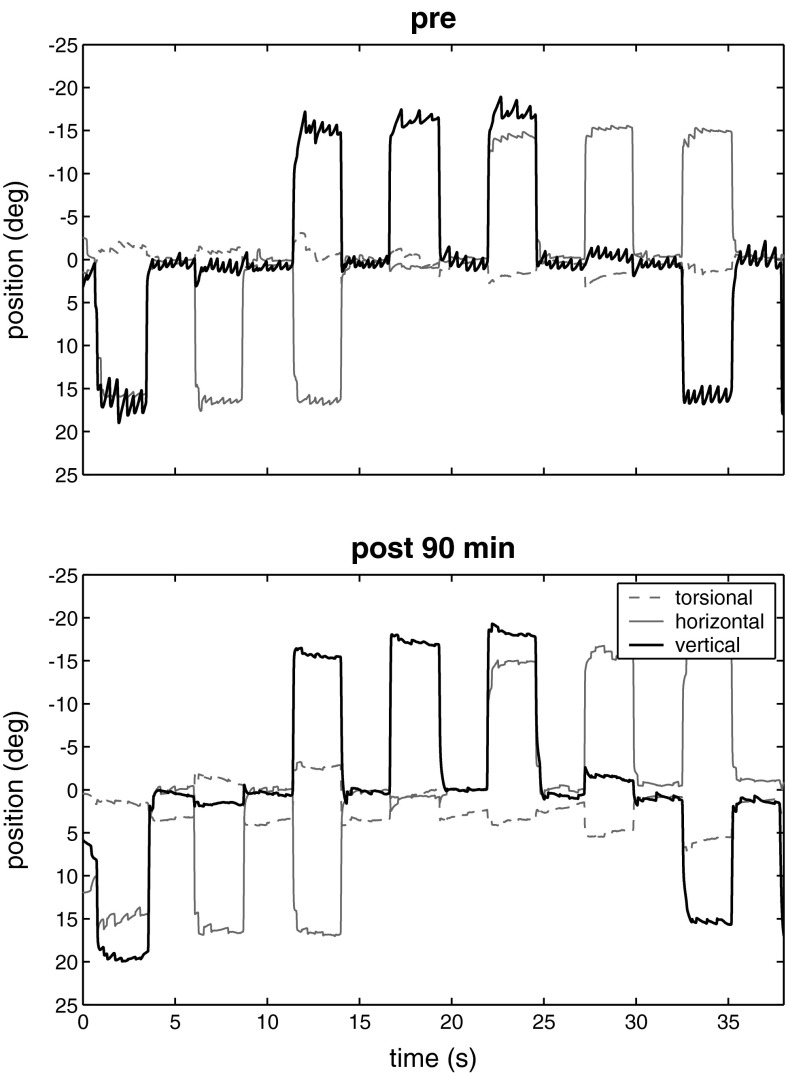
3D eye movement raw data of DBN. Vertical eye position data in three different vertical eye positions (±18°, up “–deg”, down “+deg”) while fixating a continuously visible target. The recordings were performed before and 90 min after ingestion of 10 mg 4-AP

DBN is a frequent form of acquired fixation nystagmus [8] and manifests with oscillopsia, postural instability, and cerebellar gait disorder [9, 10]. Additional ocular motor signs such as gaze-evoked nystagmus and deficient smooth pursuit eye movements are often associated with DBN and indicate a cerebellar dysfunction [9, 11, 12]. In the majority of patients, DBN is caused by a bilaterally impaired function of the cerebellar floccular lobe due to neurodegeneration [8, 13]. Different GABAergic substances have been used to treat DBN, but with only moderate success [14]. 3,4-diaminopyridine (3,4-DAP), which is a non-selective blocker of the Kv family of voltage-gated potassium channels, effectively suppresses DBN probably via an inhibition of potassium channels of Purkinje cells [15]. 4-aminopyridine (4-AP) also alleviates the symptoms of DBN [16] (Fig. 1), particularly in patients with cerebellar atrophy [17]. In a double-blind prospective crossover study, equivalent doses of 4-AP and 3,4-DAP were compared. 4-AP is more lipid-soluble, crosses the blood–brain barrier more easily, and turned out to be superior to 3,4-DAP at reducing the slow-phase velocity (SPV) of DBN [18]. Both aminopyridines were well tolerated and showed no major side effects apart from nausea, transient paresthesia, or headache. In a randomized double-blind crossover trial of 4-AP in DBN, a dosage of 5 mg four times a day reduced postural sway particularly in older patients [19]. Slow phase velocity (SPV) decreased from 2.42°/s at baseline to 1.38°/s with 5 mg 4-AP, 60 min after drug administration. Near visual acuity increased significantly from 0.59 at baseline to 0.66 with 4-AP. Using age as a covariate, increasing age correlated significantly with the 4-AP-related decrease in SPV. However, there were no differences between 4-AP and placebo regarding patient satisfaction and side effects [19]. This lack of subjective improvement may be overcome by the sustained-release form of 4-AP, which has shown its efficacy in an observational study [20]. Therefore, further trials on the sustained-release formulation are needed to confirm these results and to evaluate the long-term effects of 4-AP.

In conclusion, 4-AP in a dosage of 5 mg two to four times per day is recommended for the treatment of DBN; patients should have an electrocardiogram both at baseline and about 45 min after taking 4-AP to exclude QT interval prolongation. The sustained-release form of 4-AP, which is approved for the treatment of gait disorders in MS, can also be used in a dosage of 10 mg one to two times per day.

Chlorzoxazone (CHZ), a non-selective activator of small-conductance, calcium-activated potassium channels (SK channels), was evaluated as a potential new substance for the symptomatic treatment of DBN. This pilot study showed that CHZ 500 mg 3 times a day may improve eye movements and visual acuity [21]. Based on these findings, a randomized placebo-controlled trial is necessary.

Upbeat nystagmus (UBN) is another form of acquired vertical nystagmus, which manifests with oscillopsia due to retinal slip of the visual scene and postural instability. It usually increases with upgaze and is analogous to DBN associated with impaired upward pursuit [22]. It can be caused by lesions at several separate sites of the brain stem or the cerebellum, such as the anterior cerebellar vermis, the pontomesencephalic junction, or the medulla. Possible causes are ischemia, multiple sclerosis, tumor, cerebellar degeneration, or Wernicke’s encephalopathy [7]. The pathophysiological hypothesis describes an imbalance of the vertical vestibulo-ocular reflex tone as suggested for DBN. The competing hypothesis postulates a deficiency of the neural velocity-to-position integration of eye movements [23]. In an uncontrolled study, only moderate success was shown for the GABAergic substance baclofen (5–10 mg/3 times a day) [24]. In a single case report, 10 mg of 4-AP reduced UBN effectively and restored the impaired upward smooth pursuit, probably by improving the function of cerebellar pathways that mediate gaze holding and smooth pursuit [25]. This result needs to be corroborated in a larger cohort of UBN patients to determine the usefulness of aminopyridines in different forms of UBN. However, in contrast to downbeat nystagmus, UBN usually shows a spontaneous recovery, which makes medical treatment clinically less relevant.

There are less frequent forms of central nystagmus, which can be improved by different agents (for an overview see Table 2). Patients with periodic alternating nystagmus (PAN) often complain oscillopsia due to a horizontal jerk nystagmus, which changes its direction every 60–180 s. Like many other forms of nystagmus, PAN is often caused by cerebellar dysfunction, in particular by lesions of the nodulus or the uvula. The treatment of choice is the GABAergic drug baclofen in a dosage of 5–10 mg three times daily, which usually abolishes nystagmus in most patients [4, 5]. Patients who are refractory to a baclofen monotherapy might respond if the glutamate antagonist memantine is added [4]. Acquired pendular nystagmus (APN) is another central nystagmus, characterized by quasi-sinusoidal horizontal, vertical, or torsional components [4]. Associated symptoms, in particular oculopalatal tremor, often depend on the underlying pathology with damage to the paramedian tract projections and denervation of the inferior olive. The most common causes are multiple sclerosis, brain stem ischemia, and Whipple’s disease [5]. We recommend gabapentin (300–600 mg three times daily) or memantine (20–40 mg daily) as medical treatment. Recent reviews providing detailed therapeutic recommendations for different forms of central nystagmus are found elsewhere [4, 5].

**Table 2:** Treatment for different forms of central nystagmus, which can be improved by pharmacological agents

Pharmacological treatment of central nystagmus forms		
Nystagmus	Medication	Dosage
DBN	3,4-DAP	10 mg PO tid
	4-AP	5–10 mg PO tid
UBN	4-AP	5–10 mg PO tid
PAN	Baclofen	5–10 mg PO tid
APN	Gabapentin	300–600 mg PO tid (up to 2400 mg/d)
	Memantine	20–40 mg PO/d

*DBN* downbeat nystagmus, *UBN* upbeat nystagmus, *PAN* periodic alternating nystagmus, *APN* acquired pendular nystagmus, *3*,*4-DAP* 3,4-Diaminopyridine, *4-AP* 4-aminopyridine, *d* day, *tid* three times daily

### Vestibular migraine

Vestibular migraine is a form of migraine in which the patient experiences recurrent attacks of vertigo or dizziness [6]. More recently, the diagnostic criteria were defined more precisely by the Classification Committee of Vestibular Disorders of the Barany Society together with the International Classification of Headache Disorders (ICHD) [7]. The criteria are as follows: 1. at least five episodes with vestibular symptoms of moderate or severe intensity, lasting 5 min to 72 h; 2. current or previous history of migraine with or without aura according to the ICHD; 3. one or more migraine features with at least 50 % of the vestibular episodes: 3a. headache with at least two of the following characteristics: one-sided location, pulsating quality, moderate or severe pain intensity, aggravation by routine physical activity; 3b. photophobia and phonophobia; 3c. visual aura; 4. not better accounted for by another vestibular or ICHD diagnosis [7].

Several drugs are widely used for vestibular migraine either for the attacks or as a prophylactic treatment to reduce the frequency; however, none of them has proven their efficacy in clinical trials and the need for state-of-the-art randomized controlled trials was identified in a recent Cochrane Review [6]. There is an ongoing multicenter, double-blind, placebo-controlled trial on the prophylactic treatment of vestibular migraine with metoprolol 95 mg per day [University of Munich (PROVEMIG-trial, funded by the Federal Ministry of Education and Research)].

### Episodic ataxia type 2

EA2 is the most frequent form of inherited syndrome with recurrent attacks of vertigo and ataxia and is caused by mutations of the CACNA1A gene encoding the α-subunit of a P/Q-type calcium channel [26]. Symptoms are recurrent vertigo and ataxic symptoms lasting up to several hours, which are often elicited by stress, physical activity, or alcohol [27]. The patients often show oculomotor disturbances such as DBN, saccadic smooth pursuit, or gaze-holding deficits even outside of attacks [28], which might allow the clinican to differentiate EA2 from vestibular migraine with minor ocular motor deficits [29, 30]. About two-thirds of EA2 patients respond to the carboanhydrate inhibitor acetazolamide (250–1000 mg/d) [31], which has been the first-line treatment for many years. However, there are so far no randomized, placebo-controlled trials on the efficacy of acetazolamide. Furthermore, the side effects of acetazolamide (e.g., kidney stones, nephrocalcinosis, paresthesia, muscle stiffening with easy fatigability, hyperhydrosis) often limit its therapeutic use in clinical practice.

In 2004, a case series on three patients with EA2 showed a reduction in the number of attacks [32]. These findings were confirmed in a randomized, placebo-controlled trial in ten subjects with EA-2. During the study, the median monthly attack frequency under placebo was 6.5 and decreased to 1.65 under medication with 4-AP [33]. Furthermore, the median monthly attack duration was reduced and the quality of life as measured by the Vestibular Disorders Activities of Daily Living Scale improved.

Prior to the results of the controlled trial, animal studies in EA2 mutant mice showed that 4-AP raised the threshold for the triggering of the episodic attacks [34]. Moreover, the precision of pacemaking in Purkinje cells was restored by prolonging and increasing the action potential after hyperpolarization by targeting the K(v)1 family of K(+) channels [34]. Interestingly, the therapeutic efficacy of 4-AP was comparable and not greater than that of chlorzoxazone, which also restores the precision of Purkinje cell pacemaking. Because 4-AP in higher concentrations blocks a large array of K^+^channels, the development of more selective drugs for a safer treatment of cerebellar ataxia is necessary.

More recently, a case series showed the efficacy of the sustained-release form of 4-AP [35]. The recommended dosage of 4-AP is 5–10 mg three times a day. There is currently an ongoing placebo-controlled trials on 4-AP in EA2 investigating the sustained-release form of 4-AP versus acetazolamide versus placebo (University of Munich, EAT2TREAT, funded by the Federal Ministry of Education and Research).

### Cerebellar ataxias

Cerebellar syndromes due to neurodegenerative and hereditary diseases often lead to postural instability associated with cerebellar ocular motor disorders such as DBN, gaze-evoked nystagmus, deficient smooth pursuit, and dysmetric saccades [9]. These ocular motor deficits are leading symptoms that help to uncover mild forms of cerebellar ataxia in the absence of further deficits such as ataxia of the extremities, speech disturbances, or unsteadiness of gait.

The treatment of cerebellar motor deficits remains difficult in both recessive and dominant cerebellar ataxias. Clinical studies are often performed in genetically heterogeneous or genetically non-defined degenerative cerebellar syndromes, while large randomized-cohort studies on cerebellar symptoms are lacking, and so far no pharmacological intervention has been proven effective [36]. Recently, aminopyridines improved the motor behavior in a mouse model of spinocerebellar ataxia type 1 [37]. Those mice who were treated early in the course of the disease demonstrated better motor performance, which might be due to a neuroprotective effect mediated by an enhanced electrical activity of cerebellar Purkinje cells [37]. Currently, aminopyridines and acetazolamide may be the only effective drug for a subset of cerebellar symptoms [36]. In a retrospective case series, patients with cerebellar gait disorders due to different etiologies also benefitted from 4-AP [38]. The sustained-release form of 4-AP showed modest short-term improvements in a short-term trial with 16 patients with cerebellar ataxia (SAOA, SCA1/3/6, POLG mutation) [39]. These observations are currently being evaluated further in two placebo-controlled trials [University of Florida (NCT01811706), University of Munich (FACEG)].

**Fig. 2:**
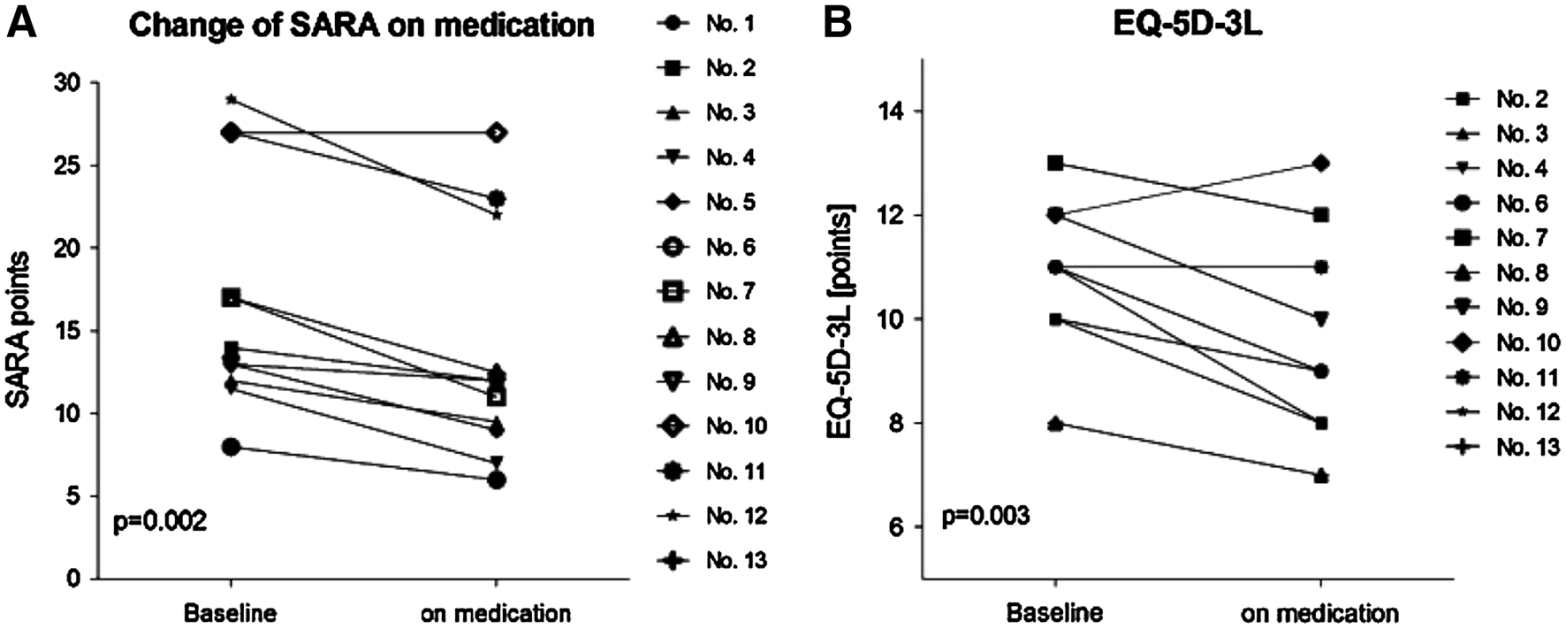
Individual changes of cerebellar patients on **a** Scale for the Rating and Assessment of Ataxia (SARA) and **b** Quality of Life Questionnaire Euro-Qol-5D-3L (EQ-5D-3L) on medication with acetyl-dl-leucine (5 g/day) for 9 ± 3 days [40]

Finally, the modified amino-acid acetyl-DL-leucine might be another drug to improve ataxia. Acetyl-DL-leucine has been used in France for more than 50 years for the symptomatic treatment of vertigo. In a case series, this substance improved ataxia (Fig. 2) and dysarthrophonia in a variety of patients suffering from cerebellar ataxia [40].

In a case series on patients with Niemann-Pick type C, acetyl-DL-leucine significantly improved ataxic symptoms without relevant side effects, thus showing a reasonable risk–benefit profile [41]. Finally, a PET study in patients with ataxia of different etiologies given AL demonstrated an increased metabolism in the midbrain and lower brain stem in responders [42].

In an animal model of acute unilateral labyrinthectomy, acetyl-DL-leucine restored the membrane potential of both depolarized and hyperpolarized vestibular neurons, presumably by interacting with membrane phospholipids such as phosphatidylinositol 4,5-bisphosphate [43, 44]. Thus, acetyl-DL-leucine may regulate the membrane potential of cerebellar Purkinje cells and therefore influence motor control and adaptive vestibular–cerebellar mechanisms. The recent clinical findings form the basis for a multi-national, placebo-controlled trial on the effects of acetyl-DL-leucine on cerebellar ataxia (ALCAT, funded by the Federal Ministry of Education and Research, first patient in: Dec 15th 2015).

## Conclusion

Recently progress has been made in the pharmacotherapy of nystagmus and cerebellar disorders. The treatment of several central vestibular disorders (DBN, UBN), and cerebellar disorders (EA2) with aminopyridines has proven to be effective. Randomized controlled trials with the sustained-release form of 4-AP in patients with cerebellar gait disorder and EA2 are on their way.

However, we need further placebo-controlled trials for new promising agents such as chlorzoxazone. In cerebellar ataxia, a prospective, randomized, placebo-controlled, multinational trial is currently being initiated to investigate the action of acetyl-DL-leucine on cerebellar symptoms (ALCAT, funded by the BMBF). Finally, more translational research will help us to find new therapeutic strategies and increase the quality of life of our patients.
